# Safety of Induced Sputum Collection in Children Hospitalized With Severe or Very Severe Pneumonia

**DOI:** 10.1093/cid/cix078

**Published:** 2017-05-27

**Authors:** Andrea N. DeLuca, Laura L. Hammitt, Julia Kim, Melissa M. Higdon, Henry C. Baggett, W. Abdullah Brooks, Stephen R. C. Howie, Maria Deloria Knoll, Karen L. Kotloff, Orin S. Levine, Shabir A. Madhi, David R. Murdoch, J. Anthony G. Scott, Donald M. Thea, Tussanee Amornintapichet, Juliet O. Awori, Somchai Chuananon, Amanda J. Driscoll, Bernard E. Ebruke, Lokman Hossain, Yasmin Jahan, E. Wangeci Kagucia, Sidi Kazungu, David P. Moore, Azwifarwi Mudau, Lawrence Mwananyanda, Daniel E. Park, Christine Prosperi, Phil Seidenberg, Mamadou Sylla, Milagritos D. Tapia, Syed M. A. Zaman, Katherine L. O’Brien, for the PERCH Study Group, Katherine L. O’Brien, Orin S. Levine, Maria Deloria Knoll, Daniel R. Feikin, Andrea N. DeLuca, Amanda J. Driscoll, Nicholas Fancourt, Wei Fu, Laura L. Hammitt, Melissa M. Higdon, E. Wangeci Kagucia, Ruth A. Karron, Mengying Li, Daniel E. Park, Christine Prosperi, Zhenke Wu, Scott L. Zeger, Nora L. Watson, Jane Crawley, David R. Murdoch, W. Abdullah Brooks, Hubert P. Endtz, Khalequ Zaman, Doli Goswami, Lokman Hossain, Yasmin Jahan, Hasan Ashraf, Stephen R. C. Howie, Bernard E. Ebruke, Martin Antonio, Jessica McLellan, Eunice Machuka, Arifin Shamsul, Syed M.A. Zaman, Grant Mackenzie, J. Anthony G. Scott, Juliet O. Awori, Susan C. Morpeth, Alice Kamau, Sidi Kazungu, Micah Silaba, Karen L. Kotloff, Milagritos D. Tapia, Samba O. Sow, Mamadou Sylla, Boubou Tamboura, Uma Onwuchekwa, Nana Kourouma, Aliou Toure, Shabir A. Madhi, David P. Moore, Peter V. Adrian, Vicky L. Baillie, Locadiah Kuwanda, Azwifarwi Mudau, Michelle J. Groome, Nasreen Mahomed, Henry C. Baggett, Somsak Thamthitiwat, Susan A. Maloney, Charatdao Bunthi, Julia Rhodes, Pongpun Sawatwong, Pasakorn Akarasewi, Donald M. Thea, Lawrence Mwananyanda, James Chipeta, Phil Seidenberg, James Mwansa, Somwe wa Somwe, Geoffrey Kwenda

**Affiliations:** 1 International Vaccine Access Center, Department of International Health, and; 2 Department of Epidemiology, Johns Hopkins Bloomberg School of Public Health, Baltimore, Maryland;; 3 Kenya Medical Research Institute–Wellcome Trust Research Programme, Kilifi;; 4 Department of Pediatric Safety, Johns Hopkins School of Medicine, Johns Hopkins University, Baltimore, Maryland;; 5 Global Disease Detection Center, Thailand Ministry of Public Health–US Centers for Disease Control and Prevention Collaboration, Nonthaburi;; 6 Division of Global Health Protection, Center for Global Health, Centers for Disease Control and Prevention, Atlanta, Georgia;; 7 Department of International Health, Johns Hopkins Bloomberg School of Public Health, Baltimore, Maryland;; 8 International Centre for Diarrhoeal Disease Research, Bangladesh (icddr,b), Dhaka and Matlab;; 9 Medical Research Council Unit, Basse, The Gambia;; 10 Department of Paediatrics, University of Auckland, and; 11 Centre for International Health, University of Otago, Dunedin, New Zealand;; 12 Division of Infectious Disease and Tropical Pediatrics, Department of Pediatrics, Center for Vaccine Development, Institute of Global Health, University of Maryland School of Medicine, Baltimore;; 13 Bill & Melinda Gates Foundation, Seattle, Washington;; 14 Medical Research Council, Respiratory and Meningeal Pathogens Research Unit, and; 15 Department of Science and Technology/National Research Foundation, Vaccine Preventable Diseases Unit, University of the Witwatersrand, Johannesburg, South Africa;; 16 Department of Pathology, University of Otago, and; 17 Microbiology Unit, Canterbury Health Laboratories, Christchurch, New Zealand;; 18 Department of Infectious Disease Epidemiology, London School of Hygiene & Tropical Medicine, United Kingdom;; 19 Center for Global Health and Development, Boston University School of Public Health, Massachusetts;; 20 Sa Kaeo Provincial Hospital, Thailand Ministry of Health;; 21 Nakhon Phanom Provincial Hospital, Nakhon Phanom, Thailand;; 22 Department of Paediatrics and Child Health, Chris Hani Baragwanath Academic Hospital and University of the Witwatersrand, Johannesburg, South Africa;; 23 University Teaching Hospital, Lusaka, Zambia;; 24 Milken Institute, School of Public Health, Department of Epidemiology and Biostatistics, George Washington University, Washington, District of Columbia;; 25 Department of Emergency Medicine, University of New Mexico, Albuquerque;; 26 Centre pour le Développement des Vaccins (CVD-Mali), Bamako; and; 27 London School of Hygiene & Tropical Medicine, United Kingdom; 28 Johns Hopkins Bloomberg School of Public Health, Baltimore, Maryland; 29 Bill & Melinda Gates Foundation, Seattle, Washington; 30 Centers for Disease Control and Prevention (CDC), Atlanta, Georgia; 31 The Emmes Corporation, Rockville, Maryland; 32 Nuffield Department of Clinical Medicine, University of Oxford, United Kingdom; 33 University of Otago, Christchurch, New Zealand; 34 ICDDR,b, Dhaka and Matlab, Bangladesh; 35 Medical Research Council, Basse, The Gambia; 36 KEMRI–Wellcome Trust Research Programme, Kilifi, Kenya; 37 Division of Infectious Disease and Tropical Pediatrics, Department of Pediatrics, Center for Vaccine Development, Institute of Global Health, University of Maryland School of Medicine, Baltimore, Maryland and Centre pour le Développement des Vaccins (CVD-Mali), Bamako, Mali; 38 Respiratory and Meningeal Pathogens Research Unit, University of the Witwatersrand, Johannesburg, South Africa; 39 Thailand Ministry of Public Health–US CDC Collaboration, Nonthaburi, Thailand; 40 Boston University School of Public Health, Boston, Massachusetts and University Teaching Hospital, Lusaka, Zambia

**Keywords:** PERCH, induced sputum, very severe pneumonia, severe pneumonia, safety.

## Abstract

**Background.:**

Induced sputum (IS) may provide diagnostic information about the etiology of pneumonia. The safety of this procedure across a heterogeneous population with severe pneumonia in low- and middle-income countries has not been described.

**Methods.:**

IS specimens were obtained as part a 7-country study of the etiology of severe and very severe pneumonia in hospitalized children <5 years of age. Rigorous clinical monitoring was done before, during, and after the procedure to record oxygen requirement, oxygen saturation, respiratory rate, consciousness level, and other evidence of clinical deterioration. Criteria for IS contraindications were predefined and serious adverse events (SAEs) were reported to ethics committees and a central safety monitor.

**Results.:**

A total of 4653 IS procedures were done among 3802 children. Thirteen SAEs were reported in relation to collection of IS, or 0.34% of children with at least 1 IS specimen collected (95% confidence interval, 0.15%–0.53%). A drop in oxygen saturation that required supplemental oxygen was the most common SAE. One child died after feeding was reinitiated 2 hours after undergoing sputum induction; this death was categorized as “possibly related” to the procedure.

**Conclusions.:**

The overall frequency of SAEs was very low, and the nature of most SAEs was manageable, demonstrating a low-risk safety profile for IS collection even among severely ill children in low-income-country settings. Healthcare providers should monitor oxygen saturation and requirements during and after IS collection, and assess patients prior to reinitiating feeding after the IS procedure, to ensure patient safety.

Induced sputum (IS) examination in immunocompromised children for *Pneumocystis jirovecii* and for suspected *Mycobacterium tuberculosis* is the standard of care [1–4]. Given its utility in this subset of children, there has been increased interest in its use for pneumonia diagnosis in children more generally [5]. While several small studies have found that the IS procedure was well tolerated in children, and produced quality specimens for pneumonia pathogen identification [2–4, 6], there have been no large-scale studies evaluating the safety of this procedure in children with severe and very severe pneumonia. We describe the safety profile of the IS procedure performed at 9 sites in 7 countries, among a heterogeneous population of children 1–59 months of age hospitalized with severe and very severe pneumonia.

## METHODS

IS specimens were collected from cases enrolled in the Pneumonia Etiology Research for Child Health (PERCH) study, a 9-site, 7-country case-control study of World Health Organization (WHO)–defined severe or very severe pneumonia in hospitalized children and community controls aged 1–59 months, to estimate the causes of pneumonia in children. The study design and methods have been described elsewhere, and training materials as well as study documents are publically available describing the standardized methods that were used across heterogeneous settings and populations [7–10]. The PERCH study protocol was approved by the institutional review board or ethical review committee at each of the study site institutions and at the Johns Hopkins Bloomberg School of Public Health. Parents or guardians of all participants provided written informed consent.

### Protocol Development

During the PERCH study design phase, experts in pediatric respiratory disease were asked to synthesize the evidence on the safety and utility of IS collection in children [5]. After considering the risks and benefits, 16 pediatric pneumonia experts endorsed the collection of IS in PERCH, particularly for detection of tuberculosis and pneumocystis pneumonia. These same experts also recommended that IS be obtained from all PERCH cases using nebulization with hypertonic saline, excepting those children with a clear contraindication, such as hypoxia [5].

Of the 9 sites (in 7 countries) selected to be part of PERCH in 2010, 2 (Kenya and South Africa) were already collecting IS specimens as part of routine clinical care from children hospitalized with pneumonia, and 1 site solely for study purposes (The Gambia). A standardized operating procedure (SOP) based on the techniques used by these sites was developed and agreed upon by all investigators [11]. To facilitate cross-site collaboration and standardization of methods, teams without experience in IS collection were trained by experienced study investigators from sites using the procedure routinely. Because 6 of the 9 sites initiated sputum collection as a new study procedure, we implemented standardized clinical monitoring for all sites to assess the procedure safety and ensure the safety of study participants [12].

### IS Procedure

The IS procedure is described in detail in accompanying papers [13, 14]. In brief, at the time of IS collection, a nebulized β-agonist was administered to enrolled PERCH cases, followed by inhaled nebulized 5% hypertonic saline (to induce expectoration). Sputum was collected using a sterile catheter passed through the nose and suction was applied to aspirate the contents of the posterior nasopharynx. Without suction, the catheter was then removed from the nose and flushed with sterile normal saline into a closed mucous trap. The specimen was collected within 24 hours of admission whenever possible, among children without contraindications (see below).

In South Africa, >1 IS specimen was routinely collected from each child to enhance the detection of *M. tuberculosis*. At other sites, an additional IS specimen for tuberculosis diagnosis was collected at the discretion of the clinical provider. Multiple specimens from the same child were not pooled, as the second specimen was collected >24 hours after the first for children in whom tuberculosis was suspected.

Contraindications to collection of IS included oxygen saturation <92% on supplemental oxygen, inability to protect airway, severe bronchospasm, seizure within the preceding 24 hours, or deemed inadvisable by the treating physician. Children who underwent IS collection were closely monitored before, during, and after the procedure. Clinical measures including oxygen saturation and oxygen requirement, respiratory rate, and consciousness level (using the alert, voice, pain, unresponsive [AVPU] scale) were recorded immediately before and after the IS procedure, as well as at 30 minutes, 2 hours, and 4 hours following the procedure. Criteria for stopping the procedure were oxygen saturation ≤88% for >60 seconds or oxygen saturation of 89%–91% for >60 seconds despite an increase in supplemental oxygen. If after a period of stabilization or rest the child’s respiratory status improved to their preprocedure baseline status, IS collection was resumed when oxygen saturation was ≥92% for 5 minutes or more. If the oxygen requirement remained greater than the requirement prior to the procedure, the IS procedure was only restarted after careful evaluation of the child’s clinical status and stability, noting possible disease progression and the magnitude of change in oxygen requirement.

### Severe Adverse Event Reporting

In collaboration with the ethical review boards that approved the study, 4 criteria were established for categorizing a change in clinical status as a serious adverse event (SAE) in PERCH cases undergoing IS specimen collection. These 4 conditions were reported as SAEs if they occurred any time between the initiating the procedure and 4 hours postprocedure: (1) death (for any reason); (2) drop in oxygen saturation by ≥5% for at least 15 minutes; (3) new onset of unconsciousness or prostration; and (4) new requirement for bronchodilator or increased frequency of bronchodilator treatment. Each site assigned an independent clinician to be the site safety monitor for the study, and when an SAE occurred, the study staff completed a report describing the event. The site safety monitor was responsible for reviewing each SAE report and determining whether the event could be attributed to a specific procedure. Relatedness was assigned as definitely, probably, possibly, and probably not. Reports were then submitted for review to the PERCH study-wide safety monitor, a physician who had no other investigator role in the PERCH study, and were forwarded to the Johns Hopkins University Institutional Review Board (IRB) and the site ethical review board(s).

The above monitoring and SAE reporting criteria were developed to safeguard children with severe respiratory illness from developing complications due to a study procedure. At least 1 study investigator from each site (usually the primary study clinician) participated in a PERCH Clinical and Epidemiology Working Group that reviewed the contraindications for IS collection, the criteria for defining SAEs, and the SAEs as they occurred.

### Statistical Analysis

We performed a descriptive analysis, calculating frequencies for categorical variables and medians and interquartile ranges for continuous variables, to compare the safety parameters at the various clinical monitoring time points. Given that the PERCH guidelines specified collection of 1 IS specimen and that children from whom a second IS specimen was collected may have differed from those with 1 IS, risks were calculated separately for the first IS and the second IS. Also, separately for the first and second IS, McNemar χ^2^ test was used to assess differences in oxygen requirement, saturation, and respiratory rate before and after the IS procedure.

## RESULTS

Over the course of a 24-month enrollment period at each site, 3802 of 4232 (90%) enrolled PERCH patients underwent a procedure for induction of sputum. Of these, 851 patients had a second procedure, though 92% of these second procedures took place in one site, South Africa. In total there were 4653 procedures. A total of 3362 patients (88%) underwent an induction of sputum procedure within 24 hours of hospital admission. For the remaining 439 with the first specimen taken after 24 hours, 337 (77%) had an initial contraindication for the procedure: 102 (23%) had specimen collection deemed inadvisable by treating clinicians; 71 (16%) had a seizure in the 24 hours before being assessed for the procedure; 64 (15%) had severe bronchospasm; 63 (14%) had oxygen saturation <92%; and 37 (8%) were unable to protect their airway. The remaining 102 (23%) participants had no specified contraindication.

**Table 1.** Characteristics of Pneumonia Etiology Research for Child Health (PERCH) Study Cases With Induced Sputum Collection

Characteristic	Children With 1 IS Specimen (n = 2951)	Children With >1 IS Specimen (n = 851)^a^	All Children With IS Specimen (n = 3802)	All IS Specimens (N = 4653)
Age				
1 mo to <6 mo	1139 (38.6)	410 (48.2)	1549 (40.7)	1959 (42.1)
6–11 mo	649 (22.0)	212 (24.9)	861 (22.6)	1073 (23.1)
12–23 mo	705 (23.9)	156 (18.3)	861 (22.6)	1017 (21.9)
24–59 mo	458 (15.5)	73 (8.6)	531 (14.0)	604 (13.0)
Severity				
Severe	2114 (71.6)	599 (70.4)	2713 (71.4)	3312 (71.2)
Very severe	837 (28.4)	252 (29.6)	1089 (28.6)	1341 (28.8)
Sex				
Female	1210 (41.0)	385 (45.2)	1595 (42.0)	1980 (42.6)
Male	1741 (59.0)	466 (54.8)	2207 (58.0)	2673 (57.4)
HIV status^b^				
Positive	112 (3.8)	93 (10.9)	205 (5.4)	298 (6.4)
Negative	2527 (85.6)	751 (88.2)	3278 (86.2)	4029 (86.6)
Unknown	312 (10.6)	7 (0.8)	319 (8.4)	326 (7.0)
HIV exposure^c^				
Exposed	267 (9.0)	353 (41.5)	620 (16.3)	973 (20.9)
Unexposed	2370 (80.3)	460 (54.1)	2830 (74.4)	3290 (70.7)
Unknown	314 (10.6)	38 (4.5)	352 (9.3)	390 (8.4)
Receiving supplemental oxygen				
At admission	500 (16.9)	702 (82.5)^a^	1202 (31.6)	1904 (40.9)
Ever^d^	808 (27.4)	767 (90.1)	1576 (41.4)	2343 (50.4)
Immediately prior to IS	524 (17.8)	577 (67.8)^e^	1101 (29.0)	1580 (34.0)
Country/study site				
Kilifi, Kenya	550 (18.6)	44 (5.2)	594 (15.6)	638 (13.7)
Basse, The Gambia	588 (19.9)	8 (0.9)	596 (15.7)	604 (13.0)
Bamako, Mali	544 (18.4)	0 (0.0)	544 (14.3)	544 (11.7)
Lusaka, Zambia	517 (17.5)	1 (0.1)	518 (13.6)	519 (11.2)
Soweto, South Africa	56 (1.9)	785 (92.2)	841 (22.1)	1626 (34.9)
Nakhon Phanom and Sa Kaeo, Thailand	190 (6.4)	1 (0.1)	191 (5.0)	192 (4.1)
Dhaka and Matlab, Bangladesh	506 (17.2)	12 (1.4)	518 (13.6)	530 (11.4)

Data are presented as No. (%).

Abbreviations: HIV, human immunodeficiency virus; IS, induced sputum.

^a^Among children with >1 IS, 92% were from the South African site where it was standard of care to place children with pneumonia on oxygen.

^b^HIV negative: negative polymerase chain reaction or enzyme-linked immunosorbent assay (ELISA) results, negative maternal test results at enrollment, or absence of evidence to indicate the child is positive in settings with limited HIV transmission (Bangladesh); HIV positive: detectable viral load or HIV seropositive if >12 months old; HIV-unknown: insufficient evidence to define HIV status.

^c^HIV exposed: HIV positive, positive ELISA results (if < 12 months) or positive maternal history (maternal history must be confirmed by maternal serology for seronegative infants <7 months); HIV unexposed: (1) documented negative maternal HIV status, (2) <7 months of age with a negative ELISA, or (3) ≥7 months with a negative ELISA result and reported, but undocumented, negative maternal history; unknown HIV exposure: insufficient evidence to define HIV exposure status.

^d^At admission or 24 hours after IS collection or 48 hours after IS collection.

^e^Children with >1 IS included in numerator if they received oxygen immediately before either IS procedure.

Thirteen SAEs were reported following the IS procedure, representing a risk of 0.34% per child undergoing the procedure (0.34% for the first IS and 0.0% for the second IS; Table 2). The most common SAE (n = 9 [69%]) was a drop in oxygen saturation that required an increase in the amount of oxygen administered or the initiation of oxygen administration. The clinical condition of 8 of these children stabilized within the 4-hour monitoring period and 1 child stabilized after a longer period (>12 hours). One child met the SAE criteria for increased need for bronchodilator nebulization. One child experienced a change in level of consciousness, which was attributed by the safety monitors to a preexisting condition and not to the procedure itself.

**Table 2.** Serious Adverse Events Reported After Initiating First Induced Sputum Procedure^a^ in Pneumonia Etiology Research for Child Health (PERCH) Study Cases

Serious Adverse Event	No.	Risk of Event per Procedure (n = 3802), %
Total	13	0.34
Drop in oxygen saturation	9	0.23
New requirement or increased need for bronchodilator	1	0.03
New onset of unconsciousness or prostration	1	0.03
Death within 4 h of initiating the IS procedure (for any reason)	2	0.05
Category of relatedness to IS procedure		
Definitely	4	0.10
Probably	1	0.03
Possibly	5	0.13
Probably not	3	0.08

Abbreviation: IS, induced sputum.

^a^No serious adverse events occurred after the second IS. Table restricted to first induced sputum procedures to avoid double counting subset of children who underwent a second IS procedure in denominator.

The 13 SAEs included 2 deaths within 4 hours after the procedure. One of these deaths, in a child 5 months of age with very severe pneumonia who developed severe respiratory distress 2 hours after the IS procedure while breastfeeding and could not be resuscitated, was categorized as “possibly related” to IS collection. Another death was determined to be “probably not related” and occurred in a 16-month-old child with very severe pneumonia who was stable for >2 hours after the IS procedure but suffered a cardiorespiratory arrest during feeding and could not be resuscitated (Table 3). All but 1 of the SAEs occurred at some point during the procedure or within 2 hours. Five SAEs occurred during the procedure, 1 occurred 30 minutes after the procedure, and 6 more occurred between 30 minutes and 2 hours post-IS. The single SAE that occurred >2 hours after the procedure is described above as the 16-month-old child who died during feeding approximately 3.5 hours after IS collection, with the death being assessed as a likely aspiration event (Table 3).

**Table 3.** Description of Serious Adverse Events Occurring Within 4 Hours of Initiating the Induced Sputum Procedure

Pneumonia Severity	Age	Sex	Adverse Event	Relatedness	Details	Outcome
Very severe	6 mo	F	Drop in oxygen saturation <92% requiring increased oxygen	Definitely	Approximately 10 min into the procedure, the child’s oxygen saturation dropped below 92% for >10 min. The child’s clinical status stabilized to stable within an hour, and the oxygen saturation stabilized at 95%–98% on room air.	Resolved
Very severe	2 mo	M	New requirement for bronchodilator	Definitely	During the procedure, child required increased bronchodilator nebulization to stabilize his oxygen saturation levels, which had been steady on supplemental oxygen prior to the procedure. Bronchodilators were administered for 15 min, and by 1 h postprocedure the child was stable, without continuing need for bronchodilators.	Resolved
Very severe	23 mo	M	Drop in oxygen saturation <92% requiring increased oxygen	Definitely	Child with cyanotic heart disease experienced a drop in measured oxygen saturation from 96% on room air to 74% while receiving nebulized hypertonic saline. Oxygen was administered, and the child was clinically stable at 4 h postprocedure. Study clinicians and the local safety monitor determined that the preprocedure oxygen saturation levels may have been noted incorrectly before initiating the IS procedure.	Diagnosis of cyanotic heart disease
Severe	3 mo	F	Drop in oxygen saturation <92% requiring increased oxygen	Definitely	Child experienced an increased oxygen requirement between 30 min and 2 h postprocedure, and a ward pediatrician recommended a switch from nasal prong O_2_ at 2 L/min to a polymask at 10 L/min based on the advice of the attending physician. By 8 h postprocedure, the child had been weaned back to nasal prong oxygen and was clinically stable.	Resolved
Very severe	3 mo	M	Drop in oxygen saturation <92% requiring increased oxygen	Possibly	A child on supplemental oxygen who had stable clinical signs for 2 hours postprocedure was taken off the ward by a guardian. When study staff located the child for the 4-h postprocedure clinical monitoring, he was found to have an oxygen saturation of 80%. Supplemental oxygen was delivered; however, the child’s guardian continued to remove the oxygen, and the child did not stabilize until 48 h after the procedure, as oxygen delivery was continuously disrupted.	Resolved
Severe	1 mo	F	Drop in oxygen saturation <92% requiring increased oxygen	Possibly	30 min after the IS procedure, the child’s oxygen saturation fell to 90% for >10 min. Supplemental oxygen was administered; the child’s clinical status resolved by 4 h post-IS with an oxygen saturation of 94%–96% on room air.	Resolved
Severe	8 mo	M	Drop in oxygen saturation <92% requiring increased oxygen	Possibly	Child’s oxygen saturation fell to 95% from 100% and work of breathing increased at 30 min post-IS. An attending physician felt the child required intubation at 1–2 h post-IS for increased work of breathing. At 4 h post-IS, the child had a respiratory rate of 62/min and an oxygen saturation of 98%. The child was extubated 7 d later.	Resolved
Severe	3 mo	M	Drop in oxygen saturation <92% requiring increased oxygen	Possibly	Oxygen saturation dropped to 67% from 95% on 2 L/min nasal prong oxygen in a child with extensive multilobar pneumonia during the NP aspiration part of the IS procedure. The procedure was stopped and the oxygen saturation normalized with continued nasal prong oxygen at 2 L/min. At 4 h post-IS, the child had a respiratory rate of 86/min and 100% oxygen saturation on 12 L/min polymask oxygen. Over the ensuing several hours the child’s respiratory status deteriorated, with increasing work of breathing and oxygen requirement leading to intubation 12 h following the procedure. The child self-extubated 6 d later.	Resolved
Severe	5 mo	F	Death	Possibly	IS collected without event in a child with 98% oxygen saturation on room air. Postprocedure respiratory rate was 78 breaths/min. During breastfeeding 1 h post-IS, the child developed severe respiratory distress and died despite resuscitation efforts 2 h after the procedure. The cause of death was assessed as a likely aspiration event.	Death
Severe	30 mo	M	Drop in oxygen saturation <92% requiring increased oxygen	Probably	During the nebulization with hypertonic saline, the child’s oxygen saturation fell to 88%–92%. Low flow oxygen was started and administered for 50 min postprocedure, without attempting to wean the child off oxygen. The child’s oxygen saturation was >92% at 2 and 4 h postprocedure on room air.	Resolved
Very severe	3 mo	M	Drop in oxygen saturation <92% requiring increased oxygen	Probably not	Child experienced a seizure 1 h after the IS procedure. The child had a seizure in the 24 h prior to IS collection, which is a contraindication for the procedure. However, this was not communicated to the PERCH physician who performed the IS procedure. The procedure was stopped during the NP suctioning because of transient desaturation to 80% with O_2_ saturation returning to 95% within 60 sec of catheter withdrawal. An hour after the IS procedure was started, the child experienced another seizure. At 4 h after the IS procedure, the child was alert with an oxygen saturation of 99% on 2 L/min nasal prong oxygen.	Resolved
Very severe	16 mo	F	Death	Probably not	Child with very severe pneumonia and early signs of malnutrition was stable more than 2 h after specimen collection. Child developed dyspnea while being fed milk by her father and could not be resuscitated. The cause of death was assessed as a likely aspiration event.	Death
Very severe	11 mo	F	New onset of unconsciousness or prostration	Probably not/ unlikely	Child was stable for 1 h after the IS procedure. 90 min after the procedure, the child developed respiratory distress immediately following feeding. Two contraindications (history of seizure and inability to protect airways) should have been noted for this case. By 4 h post-IS, the child had oxygen saturation of 85% on room air, which improved to 96% on supplemental oxygen.	Resolved

Abbreviations: IS, induced sputum; NP, nasopharyngeal; O_2_, oxygen; PERCH, Pneumonia Etiology Research for Child Health.

Children with SAEs were more likely than those without SAEs to be human immunodeficiency virus (HIV) exposed (46% of children with SAEs vs 18% of children without an SAE, *P* = .018) and to have very severe pneumonia (50% vs 29%, *P* = .062) (Table 4).

**Table 4.** Characteristics of Cases With and Without a Serious Adverse Event^a^

Characteristics	Cases without an SAE (n = 3785^b^)		Cases with an SAE (n = 13)		*P* Value^c^
Site					
Kenya	591	15.6	3	23.1	.99
The Gambia	592	15.6	2	15.4	
Mali	544	14.4	0	0.0	
Zambia	516	13.6	2	15.4	
South Africa	836	22.1	5	38.5	
Thailand	189	5.0	1	7.7	
Bangladesh	517	13.7	0	0.0	
Age					
1–5 mo	1539	40.7	7	53.8	.79
6–11 mo	858	22.7	3	23.1	
12–23 mo	859	22.7	2	15.4	
24–59 mo	529	14.0	1	7.7	
Female	1587	41.9	7	53.8	.41
Severe malnutrition^d^	504	13.4	3	23.1	.36
Bronchiolitis^e^	740	19.8	3	23.1	.92
HIV positive^f^	203	5.4	2	15.4	.24
HIV exposed^g^	614	17.9	6	46.2	.06
Very severe pneumonia	1081	28.6	7	50.0	.06
Abnormal CXR^h^	1741	53.0	9	75.0	.20

Data are presented as No. (%).

Abbreviations: CXR, chest radiograph; ELISA, enzyme-linked immunosorbent assay; HIV, human immunodeficiency virus; PCR, polymerase chain reaction; SAE, serious adverse event; SD, standard deviation; WHO, World Health Organization.

^a^Among children who underwent an induced sputum procedure.

^b^This table does not include data for 4 children who had an SAE related to lung aspirates.

^c^Calculated from logistic regression (outcome = SAE) adjusted for site and age (site is adjusted for age only and age is adjusted for site only).

^d^WHO weight-for-age *z* score < –3 SDs.

^e^Bronchiolitis reported as admission or discharge diagnoses or concurrent condition.

^f^HIV positive: detectable viral load or HIV seropositive if >12 months old.

^g^HIV exposed: HIV positive, positive ELISA results (if < 12 months), or positive maternal history (maternal history must be confirmed by maternal serology for seronegative infants <7 months).

^h^Abnormal chest radiograph defined as presence of consolidation and/or other infiltrate.

In total, 31 (0.8%) children had the first IS procedure stopped because of a drop in oxygen saturation below 88% for >60 seconds. An SAE was reported in 4 of these children (see above) and the study clinician was able to restart the IS procedure for the remaining 27 children after a period of stabilization (ie, the child’s respiratory status improved and oxygen saturation was at least 92% for ≥5 minutes). Of the 851 children undergoing a second IS procedure, 14 (1.6%) had the procedure stopped due to a drop in oxygen saturation below 88% for >60 seconds, and the procedure was reinitiated in all 14. There was no change in median oxygen requirement before and after the procedure. Five children (0.5%) required an oxygen increase of >1 L/minute immediately after the procedure, compared to their baseline status (Table 5). The percentage of children requiring supplemental oxygen before the first induced sputum procedure (n = 1091 [29.2%]) dropped to 27.3% (n = 1019) by the last clinical monitoring time point at 4 hours postprocedure. There were no differences in consciousness level in the immediate period after the procedure. However, 20 children experienced a decrease in their consciousness level when comparing immediately before IS and any point during the 4-hour monitoring period after IS. Oxygen saturation remained steady at all monitoring time points (Table 5). Clinical measures were also analyzed separately for children who had a second IS, with no clinically significant changes found in that subset of children (Supplementary Table 1).

**Table 5.** Clinical Measurements in Pneumonia Etiology Research for Child Health (PERCH) Study Cases Before and After First Induced Sputum Procedure (N = 3736^a^)

Measurement	Immediately Before	Immediately After	30 Minutes After	2 Hours After	4 Hours After
Oxygen flow, L/min^b^					
No.	1091	1048	1084	1044	1019
Median (IQR)	2.0 (2.0–2.0)	2.0 (2.0–2.0)	2.0 (2.0–2.0)	2.0 (2.0–2.0)	2.0 (2.0–2.0)
Median change from baseline (IQR)	NA	0	0	0	0
No. (%) with increase of >1 L/min from baseline	NA	5 (0.5)	5 (0.5)	8 (0.7)	8 (0.7)
Oxygen saturation, %					
No.	3349	3340	3347	3336	3324
Median (IQR)	97 (95–99)	98 (96–99)	97 (95–99)	97 (96–99)	98 (96–99)
Median change from baseline (IQR)	NA	0 (–1 to 2)	0 (–1 to 1)	0 (–1 to 1)	0 (–1 to 2)
No. (%) on supplemental oxygen^c^	1091 (32.6)	1048 (31.4)	1084 (32.4)	1044 (31.3)	1017 (30.6)
Respiratory rate, breaths/min					
No.	3513	3525	3546	3542	3534
Median (IQR)	52 (44–60)	54 (46–62)	51 (42–60)	50 (42–58)	48 (40–56)
Median change from baseline (IQR)	NA	2 (–2 to 5)	0 (–4 to 3)	–2 (–6 to 2)	–2 (–8 to 1)
No. (%) tachypneic^d^	2357 (67.1)	2561 (72.7)	2308 (65.1)	2117 (59.8)	1986 (56.2)
Consciousness level (AVPU scale)^e^					
No.	3538	3528	3532	3524	3520
No. (%) V, P, or U	10 (0.3)	10 (0.3)	12 (0.3)	9 (0.3)	7 (0.2)
No. (%) with any decrease from baseline	NA	5 (0.1)	7 (0.2)	4 (0.1)	4 (0.1)

Abbreviations: AVPU, alert, voice, pain, unresponsive; IQR, interquartile range; NA, not applicable (baseline measurement).

^a^A total of 66 children had no clinical monitoring data at any time point and have been excluded from this table.

^b^Among children on supplemental oxygen at respective monitoring time points.

^c^Two children on oxygen at 4 hours postprocedure did not have oxygen saturation data and are not included in the “n” of 1017.

^d^Tachypneic: ≥60 breaths per minute (bpm) for children <2 months, ≥50 bpm for children 2–11 months, ≥40 bpm for children 12–59 months.

^e^AVPU = alert, voice, pain, unresponsive scale to assess consciousness level. V, P, and U means not alert (A), but responsive to voice (V) or pain (P), or unresponsive (U). Numbers and percentages for AVPU exclude children whose conscious level was unknown or who were pharmacologically sedated.

## DISCUSSION

The IS procedure was generally well tolerated among a large and geographically heterogeneous group of children aged 1–59 months, hospitalized with WHO-defined severe or very severe pneumonia. Serious adverse events were very infrequent (n= 13 [0.34%]) as were the subset of SAEs that were assessed as probably, possibly, or definitely related to the IS procedure (n = 10 [0.26%]). To our knowledge, this is the first large-scale study that demonstrates a low rate of SAEs with IS collection in hospitalized children with severe or very severe pneumonia across multiple settings. This study demonstrates that IS collection can be safely performed in a large sample of children in typical resource-constrained hospital; however, vigilant monitoring for up to 2 hours postprocedure is necessary.

Our findings are consistent with the low rate of SAEs associated with IS collection shown in other studies among severely ill children [15–18]. In 2 small case series of children with community-acquired pneumonia in Finland [3] and in 2 PERCH pilot studies [6, 19], the IS procedure was found to be well tolerated and to produce sputum largely of good quality (defined as <10 epithelial cells per high-power field) that contained high frequencies of bacterial and viral pathogens.

Two post-IS deaths reported as SAEs were both temporally associated with reinitiation of feeding following the IS procedure. The death that occurred in an infant who reinitiated breastfeeding approximately 2 hours after IS collection was assessed as “possibly related” to IS collection; 2 site clinicians, the site safety monitor, and 3 clinicians from the PERCH core team, including the central safety monitor, could not exclude that IS collection may have contributed to respiratory distress. The death that occurred in a toddler during cup feeding approximately 2 hours after IS was assessed as “probably not” related to IS collection. Following IS collection, it is advisable for a clinician to reassess patients prior to reinitiating feeding to ensure that the child’s respiratory status is stable enough to tolerate oral intake.

The relatedness of the reported events to the IS procedure is difficult to conclude with confidence. All children undergoing the IS procedure were hospitalized with severe or very severe pneumonia, and many were severely ill. Distinguishing procedure-related clinical deterioration from the natural history of a child’s illness is often not possible. It is conceivable that exposing children with pneumonia to hypertonic saline nebulized solution, while provoking coughing as the intended consequence, may also in rare circumstances exacerbate the underlying illness. With the amount of coughing that is induced, respiratory fatigue, aspiration of upper airway secretions, or inadequate respiration could impair the child’s clinical status. Among the 10 events that were assessed as possibly, probably, or definitely related, 2 of the children required mechanical ventilation after IS and had prolonged hospital stays.

The clinical respiratory stability of children undergoing the procedure, as measured by oxygen saturation, oxygen requirement, respiratory rate, and consciousness level, remained remarkably unaffected by the procedure. Data from some children who experienced an SAE within the 4 hours following completion of the IS procedure are missing after the time of the event and not reflected in Table 5. Although this could bias results toward showing no change from baseline, this is unlikely to impact the interpretation of results given the small number of cases for whom data was not available because of an SAE. The oxygen requirement showed a general decreasing trend after the procedure and it is possible that IS may benefit a child with a congested chest as it loosens secretions and may improve airflow. Our findings suggest that frequent monitoring is useful up to 2 hours following the IS procedure, and that the PERCH approach was appropriate for the study and may be of use for implementation as part of clinical care in hospital environments. Because only 1 SAE was detected during the 2- to 4-hour post-IS monitoring period (among 3802 who had at least 1 IS), hospital resources may best be directed toward frequent oxygen saturation monitoring up to 2 hours postprocedure. Clinical monitoring data also suggested minimal differences at the 2- and 4-hour marks for oxygen saturation, a metric that may be readily assessable in resource-limited settings.

The rigorous clinical monitoring done for children in PERCH throughout the course of the IS specimen collection provides an opportunity to see the overall clinical effects in a large and heterogeneous study population of hospitalized children. These findings support IS as a relatively safe procedure in children with severe pneumonia. Although not statistically significant, the risk of an SAE after IS collection was higher among HIV-exposed compared with HIV-unexposed children, suggesting that a different risk-benefit assessment may apply to this subset of children. Clinicians caring for children with severe pneumonia have to consider the safety, feasibility, and utility of IS, among other factors, when considering whether the risk of the procedure is warranted. Our analysis addresses only 1 component of this decision matrix; the utility of IS in diagnosing the etiology of pneumonia in children is reported in a companion article [20]. Additional limitations in the study include potential for practice variation and incomplete monitoring data (Table 2). Despite standardized protocols and twice-yearly refresher training on all study SOPs, local differences in practice, resources, staffing, and comfort levels with the IS procedure may have resulted in procedural, monitoring, and reporting variations.

The collection of an IS specimen was well tolerated in hospitalized children aged 1–59 months with severe or very severe pneumonia who were eligible for the procedure. Due to the potential for clinical deterioration, we recommend that clinicians who perform sputum induction in severely ill children consider implementing a clinical monitoring protocol to identify and treat any complications that may arise, with close attention to oxygen saturation levels during and for 2 hours following the procedure and a clinical assessment before reinitiating feeding.

## Supplementary Data

Supplementary materials are available at *Clinical Infectious Diseases* online. Consisting of data provided by the authors to benefit the reader, the posted materials are not copyedited and are the sole responsibility of the authors, so questions or comments should be addressed to the corresponding author.

## Supplementary Material

Supplementary_Materials

## Notes

***Author contributions.*** A. N. D. performed analyses and drafted the initial manuscript. L. L. H., J. K., M. M. H., and K. L. O. provided significant guidance on the development of the manuscript. J. K. served as the study safety monitor. L. L. H., H. C. B., W. A. B., S. R. C. H., M. D. K., K. L. K., O. S. L., S. A. M., D. R. M., J. A. G. S., D. M. T., and K. L. O. conceived and designed the study and supervised study conduct. A. N. D., M. M. H., T. A., J. O. A., S. C., A. J. D., B. E. E., L. H., Y. J., E. W. K., S. K., D. P. M., A. M., L. M., D. E. P., C. P., P. S., M. S., M. D. T., and S. M. A. Z. were involved in study conduct. All authors reviewed and approved the manuscript. A. N. D. had full access to all the data in the study and had final responsibility for the decision to submit for publication.

***Acknowledgments.*** We offer sincere thanks to the patients and families who participated in this study. We thank Joan Pettit of the Johns Hopkins School of Public Health IRB for guidance in establishing the SAE reporting procedures for the study. We offer our gratitude to the members of the Pneumonia Methods Working Group and PERCH Expert Group for their time and lending expertise to assist the PERCH Study Group. We also acknowledge the work of all PERCH Contributors. See Supplementary Materials for the list of members in each group.

***Financial support.*** PERCH was supported by the Bill & Melinda Gates Foundation (grant number 48968 to the International Vaccine Access Center, Department of International Health, Johns Hopkins Bloomberg School of Public Health). J. A. G. S. was supported by a clinical fellowship from the Wellcome Trust of Great Britain (award number 098532).

***Supplement sponsorship.*** This article appears as part of the supplement “Pneumonia Etiology Research for Child Health (PERCH): Foundational Basis for the Primary Etiology Results,” sponsored by a grant from the Bill & Melinda Gates Foundation to the PERCH study of Johns Hopkins Bloomberg School of Public Health, Baltimore, Maryland.

***Potential conflicts of interest.*** K. L. O. has received grant funding from GSK and Pfizer and participates on technical advisory boards for Merck, Sanofi Pasteur, PATH, Affinivax, and ClearPath. M. D. K. has received funding for consultancies from Merck, Pfizer, and Novartis and grant funding from Merck. L. L. H. has received grant funding from Pfizer and GlaxoSmithKline. K. L. K. has received grant funding from Merck Sharp & Dohme. S. A. M. has received honoraria for advisory board membership from the Bill & Melinda Gates Foundation, Pfizer, Medimmune, and Novartis; has received institutional grants from GSK, Novartis, Pfizer, Minervax, and the Bill & Melinda Gates Foundation; and has served on speaker’s bureaus for Sanofi Pasteur and GSK. All other authors report no potential conflicts. All authors have submitted the ICMJE Form for Disclosure of Potential Conflicts of Interest. Conflicts that the editors consider relevant to the content of the manuscript have been disclosed.
