# Supplementary material for: Safety of Induced Sputum Collection in Children Hospitalized With Severe or Very Severe Pneumonia
Source: Clin Infect Dis. 2017 May 27;64(Suppl 3):S301–8. doi: 10.1093/cid/cix078 (PMC5447836; doi:10.1093/cid/cix078)
Supplement: Supplementary_Materials [file cix078_suppl_Supplementary_Materials.docx]

**Supplemental Table 1: Clinical Measurements Taken Before and After Second IS Procedure (n=851^a^)**

| **Clinical Measurements** | **Immediately before** | **Immediately after** | **30 minutes after** | **2 hours**  **after** | **4 hours**  **after** |
| --- | --- | --- | --- | --- | --- |
| **Oxygen Flow (L/min)^b^** |  |  |  |  |  |
| n | 489 | 489 | 485 | 477 | 472 |
| median (Interquartile Range, IQR) | 2.0 (2.0, 2.0) | 2.0 (2.0, 2.0) | 2.0 (2.0, 2.0) | 2.0 (2.0, 2.0) | 2.0 (2.0, 2.0) |
| median change from baseline (IQR) | n/a | 0 | 0 | 0 | 0 |
| n (%) with increase of >1 L/min from baseline | n/a | 1 (0.2) | 0 (0) | 0 (0) | 1 (0.2) |
| **Oxygen Saturation (%)** |  |  |  |  |  |
| N | 677 | 677 | 677 | 669 | 662 |
| median (IQR) | 99 (97, 100) | 100 (98, 100) | 99 (98, 100) | 99 (98, 100) | 99 (98, 100) |
| median change from baseline (IQR) | n/a | 0 (0, 1) | 0 (-1, 1) | 0 (-1, 1) | 0 (-1, 1) |
| n (%) on supplemental oxygen | 489 (72.2) | 489 (72.2) | 485 (71.6) | 477 (71.3) | 470 (71) |
| **Respiratory Rate (breaths per minute)** |  |  |  |  |  |
| N | 673 | 670 | 683 | 676 | 671 |
| median (IQR) | 46 (39, 52) | 49 (42, 56) | 46 (39, 52) | 45 (39, 51) | 44 (38, 50) |
| median change from baseline (IQR) | n/a | 3 (1, 5) | 0 (-3, 3) | -1 (-4, 2) | -2 (-5, 2) |
| n (%) tachypneic^c^ | 274 (40.7) | 362 (54) | 288 (42.2) | 258 (38.2) | 236 (35.2) |
| **Consciousness Level (AVPU Scale)^d^** |  |  |  |  |  |
| n | 685 | 685 | 684 | 678 | 674 |
| n (%) V, P, or U | 0 (0) | 2 (0.3) | 1 (0.2) | 0 (0) | 0 (0) |
| n (%) with any decrease from baseline | n/a | 2 (0.3) | 1 (0.2) | 0 (0) | 0 (0) |

^a^ A total of 66 children had no clinical monitoring data at any time point and have been excluded from this table.

^b^ Among children on supplemental oxygen at respective monitoring time points.

^c^ Tachypneic: ≥60 breaths per minute (bpm) for children <2 months, ≥50 bpm for children 2-11 months, ≥40 bpm for children 12-59 months.

^d^ AVPU= Alert, Voice, Pain, Unresponsive Scale to assess consciousness level. V, P, and U means not ‘alert (A), but responsive to voice (V) or pain (P), or unresponsive (U). Numbers and percentages for AVPU exclude children whose conscious level was unknown or who were pharmacologically sedated.

**Acknowledgements:**

***PERCH Expert Group.*** William C. Blackwelder, Harry Campbell, John A. Crump, Adegoke Falade, Menno D de Jong, Claudio Lanata, Kim Mulholland, Shamim Qazi, Cynthia G. Whitney.

***Pneumonia Methods Working Group.*** Robert E Black, Zulfiqar A Bhutta, Harry Campbell, Thomas Cherian, Derrick W Crook, Menno D de Jong, Scott F Dowell, Stephen M Graham, Keith P Klugman, Claudio F Lanata, Shabir A Madhi, Paul Martin, James P Nataro, Franco M Piazza, Shamim A Qazi, and Heather J Zar.

***PERCH Contributors:***

**Bangladesh:** Kamrun Nahar, Arif Uddin Sikdir, Sharifa Yeasmin, Dilruba Ahmed, Muhammad Ziaur Rahman, Muhammad Yunus, Muhammad Al Fazl Khan, Muhammad Jubayer Chisti, Abu Sadat Muhammad Sayeem, Shahriar Bin Elahi, Mustafizur Rahman; **The Gambia:** Michel Dione, Emmanuel Olutunde, Peter Githua, Ogochukwu Ofordile, Rasheed Salaudeen, David Parker; **Kenya:** Shebe Mohamed, Siti Ndaa, Micah Silaba, Neema Muturi, Angela Karani, Sammy Nyongesa, Anne Bett, Daisy Mugo, Salim Mwarumba, Robert Musyimi, Andrew Brent, James Nokes, David Mulewa, Joyce Sande, John Odhiambo, Joshua Wambua, Nuru Kibirige, Caroline Mulunda, Hellen Mjalla, Norbert Katira, Karen Dama, Loice Masha, Christine Mutunga, Mwanajuma Ngama, Stephen Mangi, Riziki Anthony, Mwarua Yubu, Elijah Wakili, Benson Katana, Shoboi Mgunya, Emmanuel Mumba, Benedict Mver, George Kuria, Felix Githinji, Norbert Kihuha, Boniface Jibendi, Tahreni Bwanaali, Agustus Kea; **Mali:** Nana Kourouma, Aliou Toure, Mahamadou Diallo, Breana Barger-Kamate, Mariam Samake, Seydou Sissoko, Abdoul Aziz Maiga, Mariam Samake, Toumani Sidibe, Mariam Sylla, Aziz Diakite, Bassirou Diarra; **South Africa:** Azwidihwi Takalani, Andrea Hugo, Susan Nzenze, Ndulela Titi, Mmabatho Selela, Malebo Motiane, Minah Nkuna, Nonhlanhla Tsholetsane, Sibonsile Moya, Debra Katisi, Tondani Netshishivhe, Lerato Mapetla, Gudani Singo, Simphiwe Gasa, Cece Mgenge, Nozipho Mthunzi, Nombulelo Monedi, Tanja Adams, Shafeeka Mangera, Jeannette Wadula, Peter Tsaagane, Jenifer L. Vaughan, Sakina Loonat, Martin Hale, Sugeshnee Pather, Mariëtte Middel, Siobhan Trenor, Palesa Morailane, Ntombi Maya, Rene Sterley, Charné Combrinck, Given Malete, Lerato Qoza, Grizelda Liebenberg, Hendrik van Jaarsveld, Zunaid Kraft, Lisa-Marie Mollentze, Lourens Combrinck, Tsholofelo Mosome; **Thailand:** Sununta Henchaichon, Dr. Tussanee Amornintapichet, Dr. Somchai Chuananont, Toni Whistler, Juraiporn Ratanodom, Patranuch Sapchookul, Ornuma Sangwichian, Sirirat Makprasert, Manoon Hirunsalee, Possawat Jorakate, Anek Kaewpan, Duangkamol Siludjai, Apiwat Lapamnouysup, Dr. Wantana Paveenkittiporn, Waraporn Ubonphen, Dr. Peera Areerat, Ms.Yupapan Wannachaiwong, Ms Tewa Faipet, Ms Punnat Natnarakorn, Ms Ahchanan Sacharone, Mr.Winai Makmool, Ms. Kanlaya Sornwong, Ms. Promporn Sansuriwong, Ms. Ratchanida Potiya, Ms. Wasana Hongsawong, Ms.Wipa Matchaikhen, Ms. Thatsanawan Chaiyabil, Ms.Piyapai Wannarach, Ms Chamaiporn Wadeesirisak, Mr. Yuttapong Norapet, Mattana Bangkung, Mr. Barameht Piralam, Sathapana Naorat, Anchalee Jatapai, Prasong Srisaengchai, Dr. Leonard Peruski, Ms.Dawan Phaensoongnoen, Ms.Tussaaorn Klangprapan, Ms.Narawadee Dumrongdee, Ms.Atchara Srithongkham, Mr. Piyawut Noinont, Ms. Pornthip Kamlee, Ms.Siyapa Mongkornsuk; **Zambia:** Justin Mulindwa, Musaku Mwenechanya, John Mwaba, Magdalene Mwale, Julie Duncan, Kazungu Siazele, Muntanga Mapeni, Emily Hammond; **Canterbury Health Laboratory, Christchurch, New Zealand:** Trevor P. Anderson, Joanne Mitchell, Rose Watt, Shalika Jayawardena; **The Emmes Corporation, Rockville, Maryland:** Mark Wolff, Megan Sanza, Omid Neyzari.
